# Rapid assessment of avoidable blindness for health service planning

**DOI:** 10.2471/BLT.18.217794

**Published:** 2018-09-10

**Authors:** Islay Mactaggart, Sarah Wallace, Jacqueline Ramke, Matthew Burton, Andrew Bastawrous, Hans Limburg, Muhammad Babar Qureshi, Allen Foster, Hannah Kuper

**Affiliations:** a London School of Hygiene & Tropical Medicine, Keppel Street, WC1E 7HT London, England.; b CBM International, Cambridge, England.

The World Health Organization (WHO) *Universal eye health: a global action plan 2014–2019* calls for the generation of evidence on the magnitude and causes of visual impairment as well as on eye care services, to plan towards universal eye health.^1^ The Rapid Assessment of Avoidable Blindness (commonly called RAAB) is cited in the document as a standard method for generating this epidemiological evidence.

## Description of the assessment

The rapid assessment is a standardized population-based survey method to assess the prevalence and causes of visual impairment and blindness among the population older than 49 years.^2^ This survey method also generates service indicators, including cataract surgical coverage and cataract surgical outcome; to date, it has been used in over 330 surveys across 70 countries worldwide, providing a substantial contribution of data used to calculate global blindness estimates.^2^^,^^3^

The rapid assessment of avoidable blindness was developed by the International Centre for Eye Health at the London School of Hygiene & Tropical Medicine, and evolved from the Rapid Assessment of Cataract Surgical Services.^4^ The assessment provides a simple, low-cost, open-access and epidemiologically robust method to collect data to inform eye-care programmes and policies with the aim of reducing or eliminating avoidable blindness.

This assessment has several key strengths. First, it focuses on the population older than 49 years, where blindness prevalence is highest, thereby requiring a substantially smaller sample size than all-age population-based surveys. Second, it uses simple examination techniques and has low equipment needs, and is therefore low-cost and time-efficient, while providing comparable estimates to an all-age survey.^2^^,^^5^ Third, it has an open-access data entry and analysis software that provides robust, autogenerated analysis reports of key eye health indicators disaggregated by sex and age. These data assist assessment users in eye health service planning. The standardized reports ensure that findings from these assessments worldwide and over time are comparable, and the collation of data and reports online in an open repository means that they are accessible.^6^

For quality assurance, the assessment includes a certified trainer scheme, and trainers are available throughout most of the world. To be certified as a trainer, candidates must attend a one-week, face-to-face training of trainer’s workshop and then train a rapid assessment team under the supervision of a senior trainer. The new trainers are then available to train survey teams and support them to prepare for and undertake rapid assessments and interpret the generated data.

## Updating the method

The International Centre for Eye Health is currently collaborating with Peek Vision^7^ and CBM International,^8^ with input from a wider technical and stakeholder group, to update several components of the overall method and develop the seventh version of the rapid assessment of avoidable blindness.

### Digitalization and new technology

The seventh version of the assessment will use paperless mobile data entry connected to a cloud-hosted, web-based system available via browser. Users will have the option of using Peek Acuity, a validated smartphone-based visual acuity test.^9^ Users will be able to access the system without needing to install software, maintain databases or use a particular operating system. Data will be held centrally, providing a higher degree of data quality assurance and potential for cross-survey analysis. All existing assessment indicators will be viewable and downloadable via websites, facilitating real-time views of the gathered data. Dashboard views of surveys in progress will also allow for real-time overview of survey progress and completeness. New interactive, web-based methods of visualizing the assessment’s data will be provided via a configurable web application to support data interpretation by eye health decision-makers.

### Additional information

Additional data for inclusion in the rapid assessment are also being tested. These data reflect the needs outlined in *Transforming our world: the 2030 agenda for sustainable development*,^10^ including dimensions of equity, coverage and quality. For example, new social variables will be tested to expand the monitoring of inequitable service access and to identify which population groups are being left behind. Age and sex are already collected in the existing tool, and additional measures of socioeconomic status, place of residence and disability will be field tested. If possible, the tool will allow the addition of other characteristics appropriate in a particular context, such as ethnicity or migrant status. Additional coverage indicators such as refractive error correction coverage and effective cataract surgical coverage, which combines post-operative visual outcome with coverage, will also be explored.^11^

### Use of data for planning

In practice, assessments’ data and reports are not always used in actionable eye health plans at the district or national level. A recent review of 279 surveys registered in the rapid assessment of avoidable blindness repository determined that only half of these had published the surveys’ findings.^12^

A key component of the updated seventh version of the assessment is the development of a formal planning module to support the use of the assessment’s data in creating effective eye health plans. To support this planning module, a qualitative study was recently undertaken at the International Centre of Eye Health under the supervision of a technical advisory group that includes epidemiologists, ophthalmologists, service planners and representatives of international organizations. This study included semi-structured interviews with rapid assessment users and eye health planners (two overlapping but distinct groups), a scoping review of available tools to support the use of survey data in planning and a review of a selection of current eye health plans.^13^ Key criteria for the translation of data outputs into effective planning are being synthesized from this work, and a theory of change for using the assessment to support planning is being developed and refined.

A rapid assessment of avoidable blindness planning module is currently being developed based on the outcomes of this study. The module is focused at the district planning level and will complement existing tools such as WHO’s eye care service assessment tool,^14^ which is used at the national level.^15^ The assessment’s planning module recognizes the importance of identifying a local “eye health champion”, such as a senior stakeholder in a position of sufficient authority, to engage further stakeholders across and beyond the eye health sector. The module synthesizes important practical elements of planning.^16^ For example, the module focuses on ensuring inclusion of the full range of stakeholders whose influence is required for the successful funding, approval and implementation of an eye health plan within the wider health system. Such stakeholders include the health ministry, local government, eye health staff, public health representatives, local service providers (private and government), non-eye health representatives, patient representative groups, WHO and other international organization representatives, and nongovernmental organizations. The module also emphasizes the need to incorporate stakeholders across different roles, such as decision-makers, implementers, funders and eye health service users, and to be clear on how they will be involved from the outset.

The module includes situational analysis guidance and templates, to support rapid assessment of avoidable blindness users in collating data required for effective planning beyond that provided by the assessment. The module acknowledges that collation of human resource, financial and service-use data can be challenging, and provides practical advice on both the minimum data needed to support planning and on how this data can be acquired. The module emphasizes the epidemiological gaps in the rapid assessment of avoidable blindness as a comprehensive eye health planning tool, such as how and where to incorporate data on childhood blindness or visual impairment, or on myopia in those younger than 50 years, and how to balance the assessment’s district-level data with national planning objectives.

In addition, the planning module, which will be supported by an optional additional training module that allows certified trainers to act as planning facilitators, supports users in interpreting the data to identify priorities and formulate a realistic action plan within a planning workshop. The module describes the implications of key assessment indicators, such as sex disparities or interpretation of the cataract surgical coverage. The module also provides practical guidance on organizing a planning workshop following synthesized principles from the eye health sector and the results of the key informant interviews described above.^17^

The planning module, along with other components of the seventh version of the rapid assessment of avoidable blindness, will greatly increase the capacity of this method to support eye health planning. All new features of this version will be field tested in an iterative process, with a view to formally launching the platform of the seventh version of the assessment at the end of 2019.

We anticipate that these changes will improve the relevance of the rapid assessment of avoidable blindness for the decades to come by increasing the quality of data collected and will support effective planning of programmes, at district-level, for the prevention of avoidable blindness. 
